# Chronic obstructive pulmonary disease upper airway microbiota alpha diversity is associated with exacerbation phenotype: a case-control observational study

**DOI:** 10.1186/s12931-019-1080-4

**Published:** 2019-06-07

**Authors:** Alexa A. Pragman, Katherine A. Knutson, Trevor J. Gould, Richard E. Isaacson, Cavan S. Reilly, Chris H. Wendt

**Affiliations:** 1 Department of Medicine, University of Minnesota and Minneapolis Veterans Affairs Medical Center, 1 Veterans Dr, Minneapolis, MN 55417 USA; 2 Division of Biostatistics, University of Minnesota School of Public Health, University Office Plaza, 2221 University Ave. SE - Suite 200, Minneapolis, MN 55414 USA; 3 University of Minnesota Informatics Institute, Biological Science Dean’s Office, Room 123 SnH, 6174A, 1475 Gortner Ave, St. Paul, MN 55108 USA; 4 Department of Veterinary and Biomedical Sciences, University of Minnesota, Room 205G VetS, 6187A, 1971 Commonwealth Ave, St. Paul, MN 55108 USA; 5 Minneapolis VA Health Care System, Attn: Dr. Alexa Pragman, Research Service (151), 1 Veterans Drive, Minneapolis, MN 55417 USA

**Keywords:** Pulmonary disease, chronic obstructive, RNA, ribosomal, 16S, Sputum, Microbiota, Streptococcus, *Haemophilus*, Moraxella, Phenotype

## Abstract

**Background:**

Chronic obstructive pulmonary disease (COPD) frequent exacerbators (FE) suffer increased morbidity and mortality compared to infrequent exacerbators (IE). The association between the oral and sputum microbiota and exacerbation phenotype is not well defined. The objective of this study was to determine key features that differentiate the oral and sputum microbiota of FEs from the microbiota of IEs during periods of clinical stability.

**Methods:**

We recruited 11 FE and 11 IE who had not used antibiotics or systemic corticosteroids in the last 1 month. Subjects provided oral wash and sputum samples, which underwent 16S V4 MiSeq sequencing and qPCR of 16S rRNA. Data were analyzed using Dada2 and R.

**Results:**

FE and IE were similar in terms of age, FEV_1_ percent predicted (FEV1pp), pack-years of tobacco exposure, and St. George’s Respiratory Questionnaire score. 16S copy numbers were significantly greater in sputum vs. oral wash (*p* = 0.01), but phenotype was not associated with copy number. Shannon diversity was significantly greater in oral samples compared to sputum (*p* = 0.001), and IE samples were more diverse than FE samples (*p* < 0.001). Sputum samples from FE had more *Haemophilus* and *Moraxella* compared to IE sputum samples*,* due to dominance of these COPD-associated taxa in three FE sputum samples. Amplicon sequencing variant (ASV)-level analysis of sputum samples revealed one ASV (*Actinomyces*) was significantly more abundant in IE vs. FE sputum (*p*_*adj*_ = 0.048, Wilcoxon rank-sum test), and this persisted after controlling for FEV1pp. Principal coordinate analysis using Bray-Curtis distance with PERMANOVA analyses demonstrated clustering by anatomic site, phenotype, inhaled corticosteroid use, current tobacco use, COPD severity, and last professional dental cleaning.

**Conclusions:**

FE have less diverse oral and sputum microbiota than IE. *Actinomyces* was significantly more abundant in IE sputum than FE sputum. The oral and sputum microbiota of COPD subjects cluster based on multiple clinical factors, including exacerbation phenotype. Even during periods of clinical stability, the frequent exacerbator phenotype is associated with decreased alpha diversity, beta-diversity clustering, and changes in taxonomic abundance.

**Electronic supplementary material:**

The online version of this article (10.1186/s12931-019-1080-4) contains supplementary material, which is available to authorized users.

## Background

Chronic obstructive pulmonary disease (COPD) is an inflammatory lung disorder with diverse clinical presentations. Some COPD patients experience severe or frequent exacerbations (episodic worsening of COPD symptoms), while others have a more indolent course. The reasons for this clinical variability are poorly understood. COPD phenotypes have been described in an effort to understand better the clinical heterogeneity of COPD. [1, 2] The frequent exacerbator phenotype [3, 4] (defined as the occurrence of ≥2 COPD exacerbations in a 12-month period) is clinically important because exacerbations accelerate loss of lung function, [5] decrease quality of life, [6] increase health care utilization, [7] and increase morbidity and mortality. [8, 9]

The ECLIPSE study showed that the best predictor of the frequent exacerbator phenotype over the course of a year was a history of exacerbation in the previous year (odds ratio 5.72 for subjects with ≥2 vs. 0 exacerbations). [4] Although the frequent exacerbator phenotype was more common in very severe COPD (by GOLD criteria [10]) than in less severe COPD, 22% of moderate COPD subjects and 47% of very severe COPD subjects were frequent exacerbators. [4, 11] Therefore, the frequent exacerbator phenotype is clinically relevant, easily identified, stable over time, and found at all stages of COPD severity.

Approximately 50% of COPD exacerbations are due to bacterial lung infection. [12] Acquisition of a new strain of *Haemophilus influenzae*, *Moraxella catarrhalis*, or *Streptococcus pneumoniae* in the lung microbiota is associated with an increased risk of exacerbation. [13] These pathogenic bacteria often colonize the lungs of COPD patients between exacerbations and become a component of the lung microbiota. [12] Furthermore, airway colonization with pathogenic bacteria – even in the absence of exacerbation – is associated with increased pulmonary symptoms and systemic inflammation. [14] Therefore, heterogeneity in the COPD lung microbiota and lung inflammation is a potential explanation for the frequent exacerbator vs. infrequent exacerbator phenotype.

Over the last several years, the lung microbiota of individuals with stable, exacerbation-free COPD have been described using 16S rRNA gene sequencing. [15–25] Several authors have also used prospective, observational cohorts to compare the lung microbiota of subjects with COPD both during clinical stability and later during exacerbation, [26–31] the largest studies of which are AERIS [32] and COPDMAP. [33] Several studies note an increase in Proteobacteria during exacerbations compared to periods of clinical stability, [29, 31–33] with some studies specifically citing *Moraxella* [31–33] and *Haemophilus* [32] as the genera responsible for this increase. While alpha diversity appears to decrease with a decline in FEV_1_, there is no consensus on whether or not alpha diversity changes at the time of exacerbation. [31–33] In particular, the COPDMAP study [33] noted differences in alpha diversity patterns across their three study centers, raising the possibility that local practice patterns in the management of COPD exacerbations may influence alpha diversity. The AERIS study [32] also described how alpha diversity and changes in taxonomic abundance are associated with severity of lung obstruction, but did not detect a statistically significant association between alpha diversity and exacerbations status. There were also limited associations between exacerbation status and taxonomic abundance.

Despite these contributions to our understanding of the COPD exacerbation microbiota, several gaps remain in our understanding of how exacerbation phenotype may correlate with the lung microbiota. Firstly, no studies have prospectively identified frequent vs. infrequent exacerbators for further examination. Secondly, we lack a complete understanding of the “stable” lung microbiota (the microbiota present during periods of clinical stability, without recent exposure to microbiota-altering drugs such as systemic corticosteroids or antibiotics) in frequent vs. infrequent exacerbators. Lastly, it is not clear to what extent sputum, which is contaminated by the oral microbiota during expectoration, can adequately reflect the lung microbiota or differentiate between disease phenotypes.

Therefore, we have undertaken the present study to address these three knowledge gaps. This case-control observational study describes the oral and sputum microbiota in relationship to exacerbation phenotype and other relevant clinical characteristics. Our central hypothesis is that the oral and sputum microbiota are important correlates or causes of exacerbations. Any potentially-modifiable clinical factors that correlate with the frequent exacerbator microbiota may suggest future targets for therapeutic intervention.

## Methods

### Subjects

Twenty-two subjects with COPD (11 frequent and 11 infrequent COPD exacerbators, referred to as FE and IE, respectively) who were over the age of 40, with a minimum of 10 pack-year history of smoking were recruited at the Minneapolis VA Medical Center. FEs were identified based on a history of ≥1 exacerbation in the last 12 months, whereas IEs were free from exacerbations for the last 24 months. All FE study participants met the GOLD criteria for FEs (at least 2 exacerbations in the prior year). [10] All participants had not used antibiotics or oral corticosteroids within the previous month. For full inclusion and exclusion criteria see Additional file 1: Table S1.

### Sample acquisition

Oral wash and sputum samples were obtained and processed as described in the supplementary information available online. Three subjects (2 IE and 1 FE) were unable to provide a sputum sample.

### DNA extraction and 16S rRNA gene sequencing

Samples and controls were extracted using the MO BIO PowerSoil DNA Isolation Kit as described in the Additional file 1: supplemental information.

### Quantitative PCR (qPCR)

To determine 16S rRNA gene copy numbers, qPCR was performed in triplicate for all samples and controls as previously described [20] and detailed in the Additional file 1: Supplemental information. Copy numbers were normalized to sample mass, which accounts for both sample volume and density.

### Data processing

**Fig. 1:**
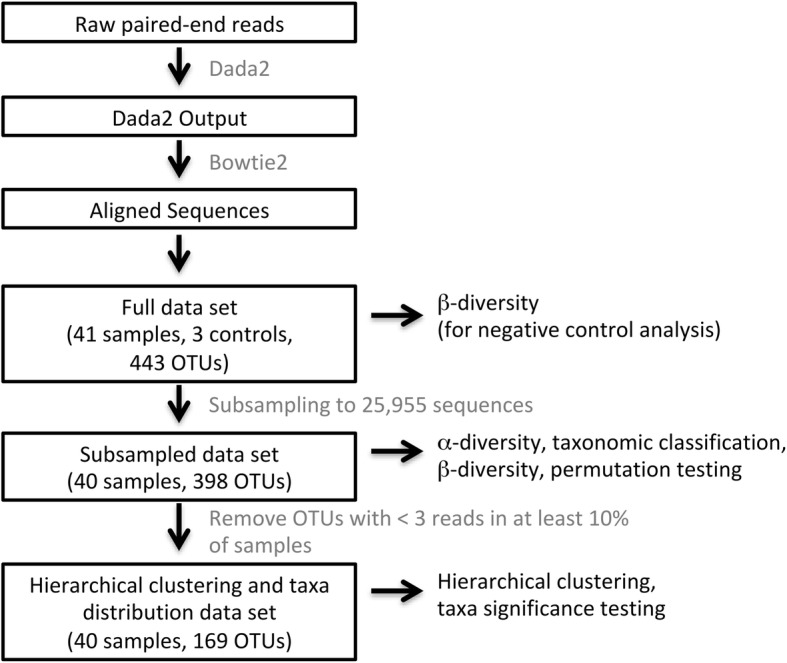
Data processing flow chart. DNA sequences were processed into data sets using the software tools and procedures as described in the text and illustrated here as a pipeline. Boxed black text indicates significant steps in the pipeline or data sets produced for specific analyses. Gray text specifies the software tools or procedures used in the pipeline. Unboxed black text specifies the analyses performed on the indicated data sets

Dada2 was used to filter, trim, dereplicate, merge paired reads, and remove primers, phix, and bimeras. [34] Bowtie2 [35] was used to remove human sequences prior to aligning sequences using the Ribosomal Database Project (RDP) Classifier [36] with the SILVA database. [37] The full data set underwent β-diversity analysis with Bray-Curtis dissimilarity to visualize control and sample similarities and determine subsampling depth. Subsampling to 25,955 sequences eliminated all negative control samples and one FE sputum sample. This subsampled data set, which consisted of 398 amplicon sequencing variants (ASVs), was used in subsequent analyses. Alpha diversity indices (Shannon and Simpson indices) were calculated using vegan while β-diversity analyses utilizing the Bray-Curtis dissimilarity were performed using phyloseq. Prior to hierarchical clustering and taxa distribution analyses, additional filtering was performed to remove ASVs that did not have at least 3 reads in 10% of all samples. 169 ASVs remained in this data set. Methods of accounting for and correcting for oral contamination of sputum samples during expectoration were considered but not undertaken to avoid introducing bias (see Additional file 1: Supplemental information for a discussion of this issue). A data processing flow chart is provided in Fig. 1.

### Statistical analysis

Subject characteristics were analyzed with a Fisher-Pitman permutation test for continuous variables and Fisher’s exact test for categorical variables. ANOVA analyses with post-hoc pairwise permutation testing were performed to compare 16S copy numbers across sites. A linear mixed model was used to evaluate for associations between 16S copy numbers, phenotype, and sampling site; *p*-values were obtained by permutation testing. α-diversity associations were tested using linear mixed models with α-diversity score as a response, patient as a random effect, and site, phenotype and their interaction as fixed effects. To determine clinical factors associated with α-diversity, PERMANOVA testing with default parameters was performed on the Bray-Curtis dissimilarity matrix. Wilcoxon rank-sum permutation tests were used to determine differences in ASV counts between phenotypes. Results were also assessed after controlling for FEV_1_ percent predicted (FEV1pp), a potential co-linear clinical variable with phenotype. ASV count data was used to cluster samples with complete linkage hierarchical clustering using hclust function in base R. All analyses were performed in R version 3.4.2. Additional statistical analysis methods are found in the Additional file 1: Supplemental information.

## Results

### Characteristics of the study participants

Subjects were all male and no differences were observed between FEs and IEs based on age, significant medical comorbidities, COPD severity, tobacco exposure, or SGRQ score. FEs, all of whom met the GOLD criteria for frequent exacerbators, [10] experienced a mean of 2.91 exacerbations in the prior year (range: 2–5 exacerbations, median: 3 exacerbations, Table 1). Three subjects were unable to provide sputum samples and one sputum sample was eliminated due to low sequencing yield. Eighteen sputum samples (from 9 FEs and 9 IEs) were available for analysis. Subjects with evaluable sputum samples did not differ significantly from the study population as a whole (Additional file 1: Table S2).

**Table 1:** Subject Characteristics

	Infrequent Exacerbator	Frequent Exacerbator	*p* value*
N	11	11	
Sex, Male (%)	11 (100)	11 (100)	
Age, mean (sd)	68.36 (5.45)	70.82 (7.12)	0.39
Race, white (%)	11 (100)	10 (90.9)	1.00
BMI, mean (sd)	29.25 (6.01)	26.09 (6.85)	0.26
Hypertension, Yes (%)	6 (54.5)	6 (54.5)	1.00
Diabetes, Yes (%)	2 (18.2)	3 (27.3)	1.00
COPD Severity (%)			
Moderate	5 (45.5)	3 (27.3)	
Severe	4 (36.4)	3 (27.3)	
Very Severe	2 (18.2)	5 (45.5)	
FEV_1_% predicted	46.82 (13.93)	35.5 (15.18)	0.088
COPD exacerbations in the last 12 months, mean (sd)	0 (0)	2.91 (1.04)	< 0.001
COPD hospitalizations in the last 12 months, mean (sd)	0 (0)	0.55 (0.69)	0.035
Inhaled corticosteroid use, Yes (%)	6 (54.5)	7 (63.6)	1.00
Pack-years of smoking, mean (sd)	61.18 (31.94)	69.55 (48.57)	0.66
Current tobacco use, Yes (%)	5 (45.5)	3 (27.3)	0.66
Current alcohol use, Yes (%)	7 (63.6)	9 (81.8)	0.64
Brush teeth ≥ once daily (%)	9 (81.8)	10 (90.9)	1.00
History of periodontal disease, Yes (%)	3 (27.3)	2 (18.2)	1.00
SGRQ Score, mean (sd)	49.72 (7.92)	48.27 (16.20)	0.792

BMI, body mass index; COPD, chronic obstructive pulmonary disease; FEV1% predicted, forced expiratory volume in 1 s, percent of predicted value; sd, standard deviation; SGRQ, St. George’s Respiratory Questionnaire

*A Fisher-Pitman permutation test was conducted for all continuous variables and a Fishers exact test for all categorical variables

### 16S rRNA copy number is associated with site, but not exacerbation phenotype

**Fig. 2:**
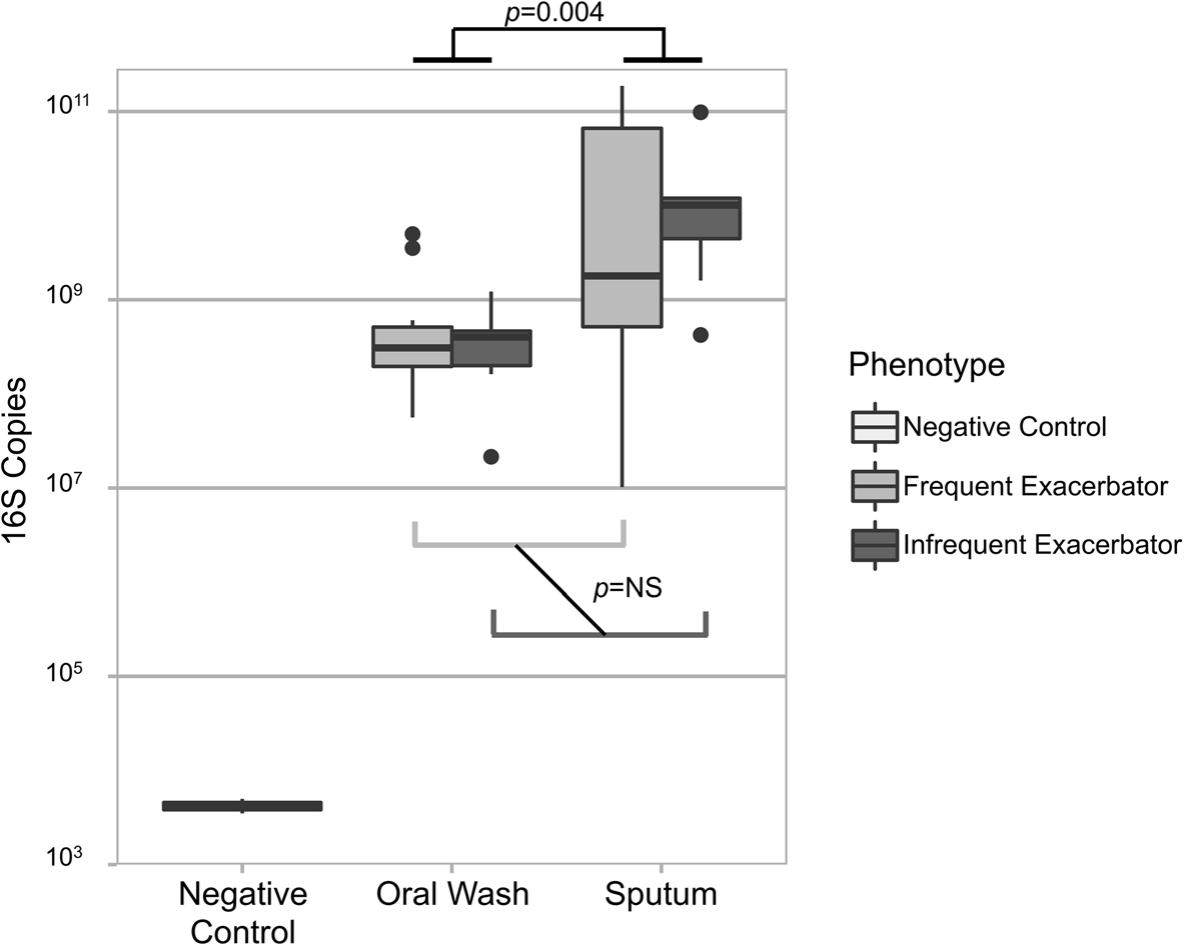
Exacerbation phenotype is not associated with bacterial biomass. 16S rRNA gene copies were determined using quantitative PCR. Negative control samples contained significantly fewer 16S copies than subjects’ oral wash and sputum samples. Sputum contained a greater number of 16S rRNA gene copies than did oral wash samples (linear mixed model with Bonferroni-Holm correction, *p* = 0.004). Exacerbation phenotype was not associated with 16S rRNA gene copy number changes, although frequent exacerbator sputum samples exhibited greater variability than infrequent exacerbator sputum samples

16S rRNA copy numbers were determined for each subject and control sample and normalized to sample mass. Sputum samples contained a greater number of 16S rRNA copies (mean 3.04 × 10^10^) than both oral wash samples (mean 7.17 × 10^8^) and negative control samples (mean 4.21 × 10^3^; ANOVA *p* < 0.001). *Post-hoc* pairwise testing for all sample pairs indicated significant differences across all pairs (all *p*-values < 0.01). Although anatomic site was a significant predictor of 16S copy numbers (*p* = 0.004), exacerbation phenotype and the interaction of anatomic site and exacerbation phenotype were not significant (Fig. 2).

### Samples are distinct from negative controls

Samples contained 17,567–136,275 reads (mean 89,961.7, median 90,420); negative controls contained 222–732 sequences (mean 417.7, median 229). The full data set underwent α-diversity analysis with Bray-Curtis dissimilarity to visualize control and sample similarities and determine subsampling depth (Additional file 1: Figure S1). Negative control samples as well as sequencing center controls clustered together, while the majority of subject samples were distributed elsewhere. Subsampling to 25,955 sequences eliminated all negative control samples and one FE sputum sample.

### Alpha diversity is associated with site and exacerbation phenotype

**Fig. 3:**
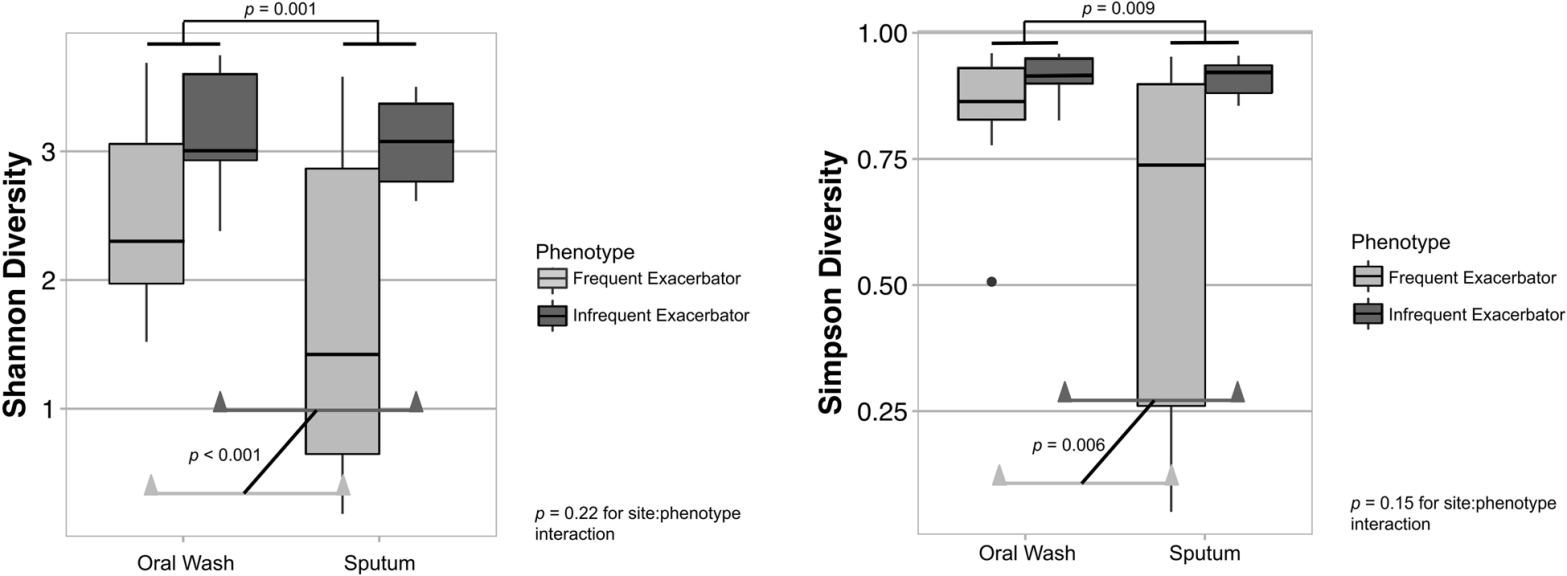
Alpha diversity is associated with exacerbation phenotype. Linear mixed models were constructed with diversity score as the response variable; site, phenotype, and their interactions as fixed effects; and subject as a random effect. **a**. Sputum samples had lower Shannon diversity scores than oral wash samples (*p* = 0.001). Frequent exacerbators had lower Shannon diversity scores than infrequent exacerbators (*p* < 0.001). The interaction of site and phenotype was 0.22. **b**. Sputum samples had lower Simpson diversity scores than oral was samples (*p* = 0.009). Frequent exacerbators had lower Simpson diversity scores than infrequent exacerbators (*p* = 0.006). The interaction of site and phenotype (*p* = 0.15) was not statistically significant, although there was some evidence for a difference. These potential associations warrant further study

Shannon and Simpson diversity indices, which incorporate both microbial richness and evenness, were determined for each subject sample. Regardless of exacerbation phenotype, alpha diversity indices were lower for sputum samples than for oral wash samples (mean oral wash Shannon diversity 3.05 vs. mean sputum diversity 2.45; mean oral wash Simpson diversity 0.90 vs. mean sputum diversity 0.76). We used a linear mixed model with anatomic site, phenotype, and their interaction as fixed effects (subject as random effect) to determine relationships between alpha diversity, anatomic site, and exacerbation phenotype. Utilizing Shannon diversity, we found that both anatomic site (*p* = 0.001) and exacerbation phenotype (*p* < 0.001) were significant predictors of Shannon diversity, with FE exhibiting significantly lower Shannon diversity than IE at both sites. There was no evidence for an interaction between site and phenotype (*p* = 0.22, Fig. 3a).

The linear mixed effects model using Simpson diversity demonstrated that anatomic site was a significant predictor of Simpson diversity (*p* = 0.009), as was exacerbation phenotype (*p* = 0.006). The interaction of site and phenotype (*p* = 0.15) was not statistically significant, although there was some evidence for differences (Fig. 3b). In both Shannon and Simpson linear models of alpha diversity, anatomic site was a more influential predictor of alpha diversity than was exacerbation phenotype or their interactions (Table 2).

**Table 2:** Alpha Diversity Effect Estimates Using a Linear Model

Factor	Shannon Diversity Model		Simpson Diversity Model	
	*p*-value	Effect Estimate	*p*-value	Effect Estimate
Anatomic Site	0.001	−0.86	0.009	−0.20
Exacerbation Phenotype	< 0.001	0.31	0.006	0.038
Interaction of Site and Phenotype	0.22	0.76	0.15	0.19

Our previous work on the COPD lung microbiota identified age as a significant predictor of α-diversity. [17, 38] We therefore included age as a predictor in our alpha diversity linear mixed models. With site, phenotype, and their interactions included in the model, age was not a significant predictor of either Shannon or Simpson diversity (data not shown).

**Fig. 4:**
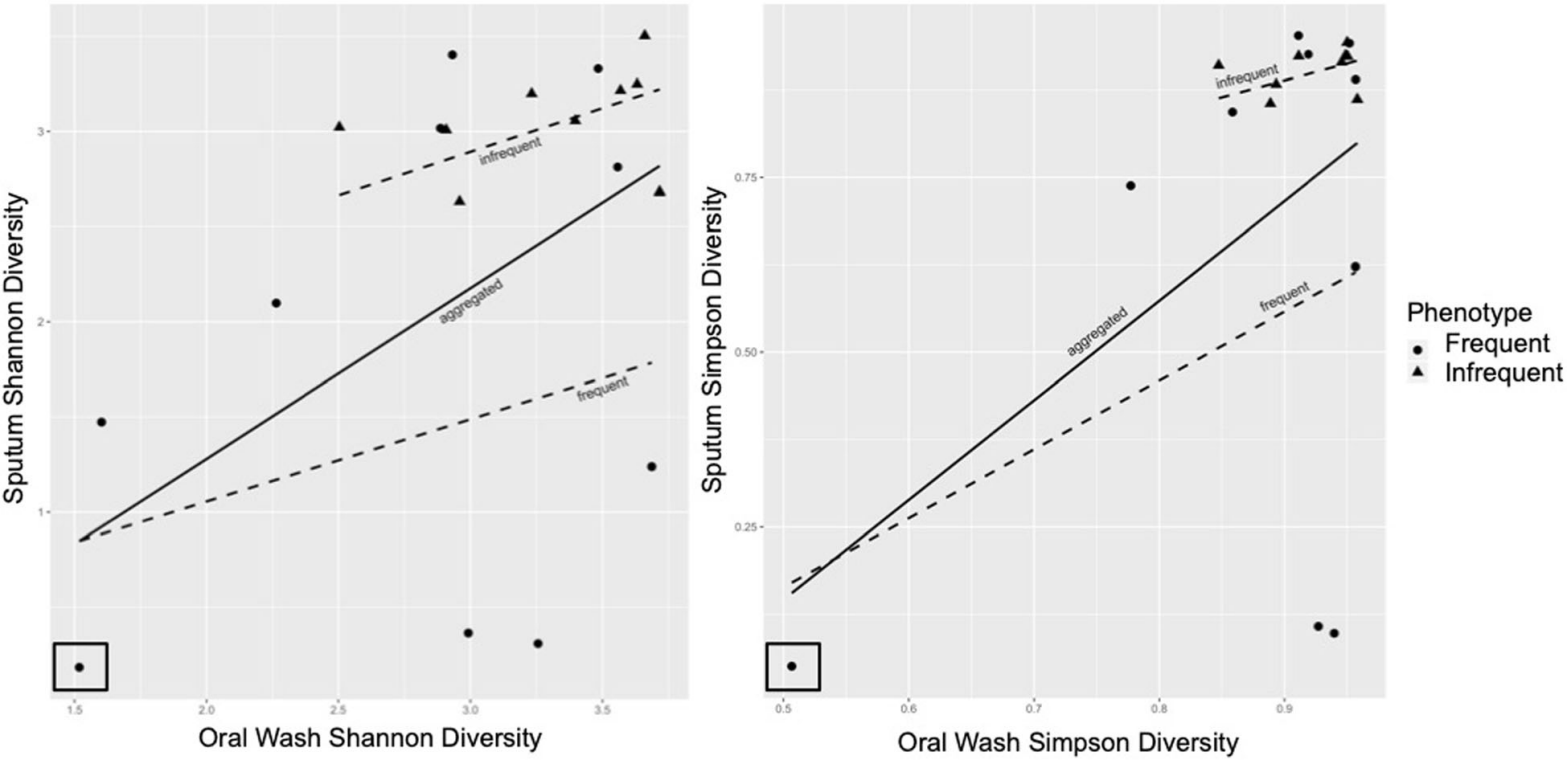
Oral Wash Alpha Diversity Is Associated with Sputum Alpha Diversity. Circles represent FE samples while triangles represent IE samples. Linear models were used to predict sputum alpha diversity based on oral wash alpha diversity. The solid lines represent the aggregated model incorporating both phenotypes while the dotted lines represent the models of frequent or infrequent exacerbator samples alone. **a**) Oral wash Shannon diversity alone was a significant predictor of sputum Shannon diversity (*p* = 0.0002), but addition of phenotype and the interaction of oral wash Shannon diversity and phenotype to the model resulted in a near-significant oral wash result (*p* = 0.07) and non-significant results for phenotype and their interactions. **b**) Oral wash Simpson diversity alone was also a significant predictor of sputum Simpson diversity (*p* = 0.0003), and incorporation of phenotype and its interaction resulted in significant results for oral wash diversity (*p* = 0.008), but non-significant results for phenotype and its interaction with oral wash diversity. The removal of the low diversity x-outlier (boxed in both panels, see text and Additional file 1: Figure S2) resulted in non-significant results for all but oral wash Shannon diversity alone as a predictor of sputum Shannon diversity (*p* = 0.039)

Sputum samples are subject to oral admixture during expectoration, and this may be a particular concern in subjects (such as IE) who may produce less sputum. We sought to understand whether there is a correlation between oral wash and sputum α-diversity in the same subject. In linear models with only oral wash α-diversity as a predictor of sputum α-diversity, both Shannon and Simpson oral wash scores were significant predictors of Shannon (*p* = 0.0002) and Simpson (*p* = 0.0003) sputum diversity, respectively (Fig. 4). We expanded these linear models to include exacerbation phenotype and the interaction of oral wash α-diversity and phenotype as predictors of sputum α-diversity. Inclusion of these additional factors in the Shannon diversity model showed that oral wash Shannon diversity alone narrowly missed significance (*p* = 0.07), while phenotype alone and the interaction of oral wash diversity and phenotype were non-significant. In the Simpson diversity model, oral wash diversity alone remained significant (*p* = 0.008), while phenotype alone and their interactions were non-significant. Close inspection of the figure, particularly the Simpson diversity model, reveals the presence of a low diversity x-outlier (boxed). Removal of this x-outlier did not significantly change the findings of the Shannon diversity model (oral wash alone remained significant [*p* = 0.039], while all factors were non-significant in the full model). However, removal of this low diversity x-outlier from the Simpson model did result in all factors becoming non-significant (oral wash alone and in combination with phenotype and their interactions; Additional file 1: Figure S2). This suggests that sputum α-diversity likely is associated with oral wash alpha diversity, but this potential relationship does not appear to be robust.

### Upper airway microbiota sites exhibit greater within-subject than between-subject similarity

**Fig. 5:**
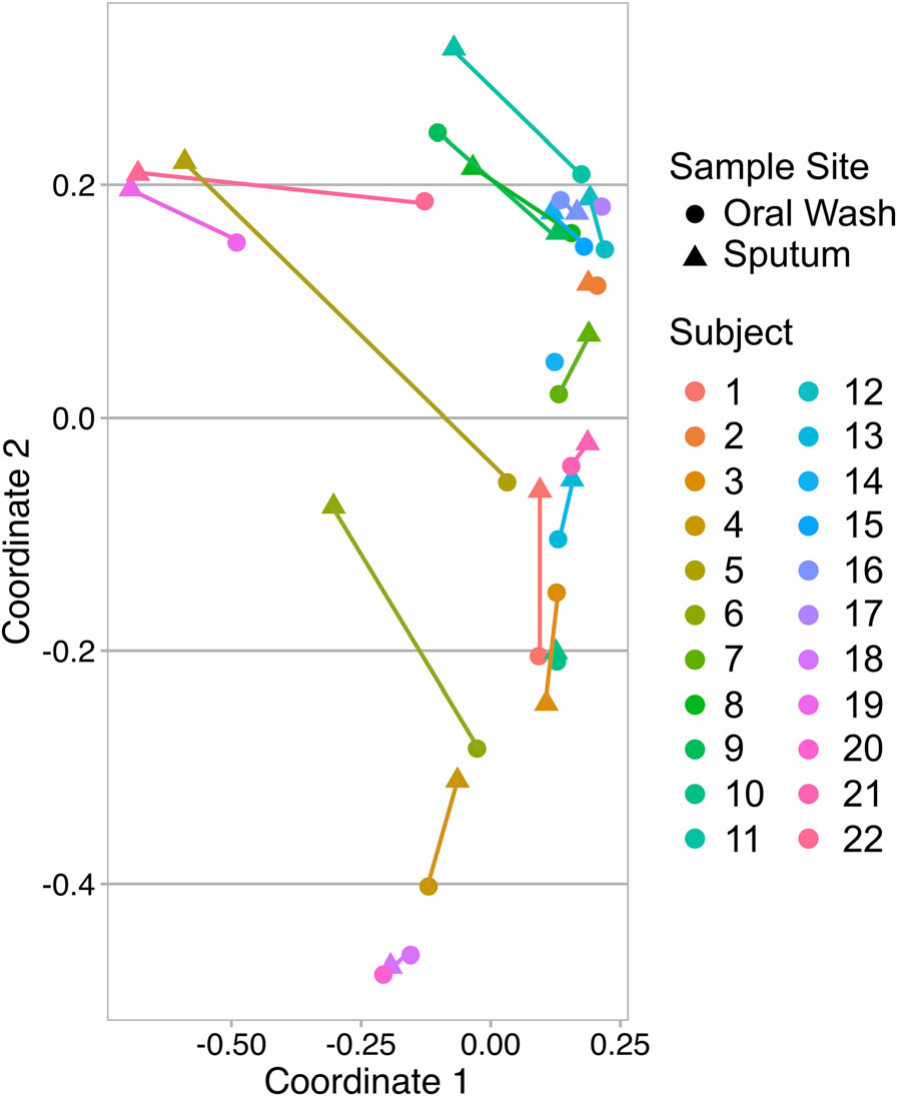
Within-subject similarity is greater than between-subject similarity. Principal coordinate analysis using Bray-Curtis dissimilarity was plotted with color used to define each subject. Each subject’s oral wash (circle) and sputum (triangle) samples are connected by a colored, subject-specific line, which defines the within-subject oral wash-sputum similarity. Four subjects did not have sputum samples available or evaluable. Permutation testing demonstrated greater within-subject than between-subject similarity at these two sites (*p* < 0.001). Exacerbation phenotype was not significantly associated with changes in within-subject similarity (*p* = 0.95)

The subsampled dataset underwent PCoA analysis using Bray-Curtis dissimilarity (Fig. 5). Univariate PERMANOVA analyses were used to identify significant clustering based on anatomic site sampled, exacerbation phenotype, COPD severity (by GOLD category), time since last professional cleaning (in the last 6 months, 6–12 months ago, or more than 12 months ago), current tobacco use, inhaled corticosteroid use, and current alcohol use. All PERMANOVA analyses except current alcohol use were statistically significant, with each variable associated with 4.3–10.4% of the variance on univariate analysis (Table 3). Time since last professional dental cleaning was associated with a larger variance than both anatomic site and exacerbation phenotype. While there was homogeneity of dispersion in beta diversity with respect to anatomic site and COPD severity, exacerbation phenotype (as well as ICS use and current tobacco use) exhibited heterogeneity of dispersion on beta diversity analysis. This suggests that FE and IE microbiotas are not homogeneous with respect to beta diversity variation.

**Table 3:** Beta Diversity is Associated with Multiple Clinical Factors on PERMANOVA Analysis

Factor	Univariate PERMANOVA		
	*p*-value	r^2^	Homogeneity of multivariate dispersions
Anatomic Site	0.016	0.091	Homogeneous (*p* = 0.225)
Exacerbation Phenotype	0.025	0.050	Heterogeneous (*p* = 0.037)
Inhaled Corticosteroid Use	0.045	0.043	Heterogeneous (*p* = 0.005)
Current Tobacco Use	0.024	0.051	Heterogeneous (*p* = 0.005)
COPD Severity	0.003	0.096	Homogeneous (*p* = 0.89)
Last Professional Dental Cleaning	0.006	0.104	Heterogeneous (*p* = 0.003)

To understand the degree of similarity between subjects’ paired oral washes and sputum samples, we compared within-subject and between-subject sample similarities. A permutation test comparing the within-subject and between-subject similarities resulted in a *p*-value of < 0.001, indicating greater within-subject than between-subject similarities at these sites (Fig. 5). We also compared the within-subject similarities for FE to the within-subject similarities of IE using a permutation test. This p-value was 0.95, indicating that within-subject oral and sputum similarities are not significantly modified by exacerbation phenotype.

**Fig. 6:**
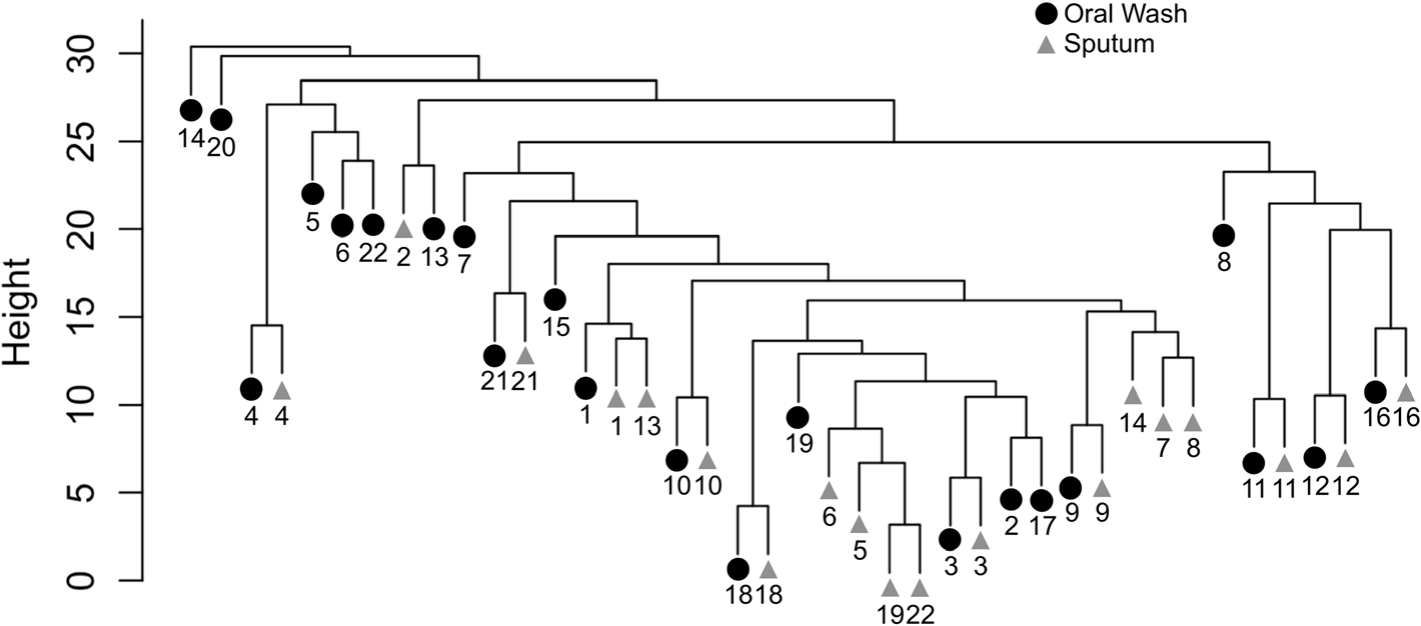
Hierarchical clustering illustrates within-subject similarity. Oral samples (circles) and sputum samples (triangles) are represented on this clustering diagram, with subject ID provided at bottom. With the exception of samples from frequent exacerbators 5, 19, and 22, subject ID (rather than sample type or exacerbation phenotype) was the primary driver of clustering. The exceptions to this were FE subjects 5, 19, and 22, whose sputa were dominated by *Haemophilus* or *Moraxella* while their oral wash samples were not dominated by these taxa. No sample pairs from IE subjects exhibited such large separations between samples

**Fig. 7:**
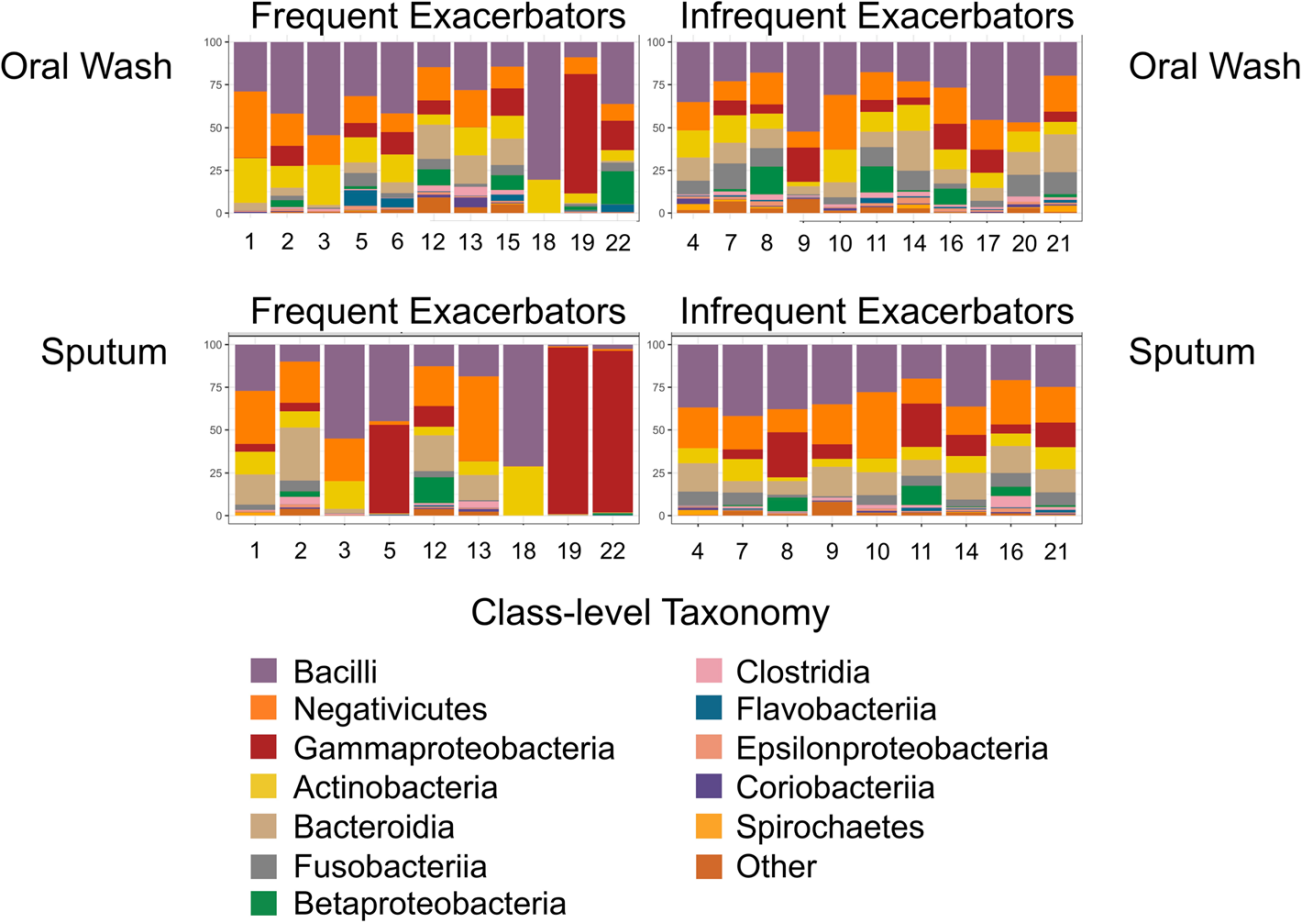
Select frequent exacerbator sputum samples differ markedly from their paired oral wash samples. Oral wash samples are presented in the top row, with sputum samples in the bottom row. Frequent exacerbator samples are present in the left column with infrequent exacerbator samples at right. Samples are labeled by subject ID to facilitate comparisons between sites for the same subject. Class-level taxonomy is provided by color coding (see legend). Oral wash samples are similar across both phenotypes, with the most common taxa being *Streptococcus* (Bacilli—purple), *Veillonella* (Negativicutes—orange), and *Haemophilus* (Gammaproteobacteria—red). Sputum samples differ visually between frequent and infrequent exacerbators, with *Haemophilus* and *Moraxella* (both Gammaproteobacteria—red) dominating frequent exacerbator sputum samples 5, 19, and 22. This marked shift in select sputum samples is neither seen across all frequent exacerbator sputum samples nor in any infrequent exacerbator samples. Comparison of frequent exacerbator paired oral wash and sputum samples reveals that this *Haemophilus* or *Moraxella* dominance is not reflected in the oral wash samples

Hierarchical clustering was used to illustrate similarities between samples. Prior to clustering, taxa with fewer than 3 reads in 10% of samples were removed from the dataset, which left 169 ASVs for analysis. The individual subject, rather than sample type or exacerbation phenotype, was the primary driver of clustering in this analysis as well (Fig. 6). The exceptions to this were FE subjects 5, 19, and 22, whose sputa were dominated by *Haemophilus* or *Moraxella* while their oral washes were not (see next section and Fig. 7). We reviewed these subjects’ clinical characteristics to determine if they otherwise differed from the FE group as a whole. All three subjects were using ICSs, which have been associated with microbiota changes, [39] while 7 of the 11 FEs (64%) were using ICSs. These three subjects’ mean FEV1pp was 34%, mean SGRQ score was 47.45, and mean number of exacerbations per year was 3. These values were similar to the group of FEs as a whole, indicating that these subjects with low diversity did not appear to differ from the other FEs with respect to disease severity.

### Exacerbation phenotype is associated with taxa changes in the sputum microbiota but not in the oral microbiota

To understand taxa shifts associated with exacerbation phenotype, we compared ASVs separately within each site. Of the 169 ASVs in the hierarchical clustering and taxa significance dataset, many were found in only one phenotype. Among oral wash samples, 43 ASVs were found only in FE, while 102 ASVs were found only in IE. Among sputum samples, 34 ASVs were found only in FE, while 101 ASVs were found only in IE. The three most common taxa in oral wash samples did not change based on subject phenotype. In contrast, the most common taxa in sputum samples differed by phenotype. *Haemophilus* was the most common genus in FE sputum samples, but the 4th most common in IE sputum samples. *Prevotella* was the 3rd most common genus in IE sputum samples and the 6th most common in FE sputum samples (Fig. 7, Table 4)*.*

**Table 4:** Taxa Abundance by Anatomic Site and Exacerbation Phenotype

Rank*	Oral Wash		Sputum	
	Frequent Exacerbator	Infrequent Exacerbator	Frequent Exacerbator	Infrequent Exacerbator
1	*Streptococcus*	*Streptococcus*	***Haemophilus***	*Streptococcus*
2	*Veillonella*	*Veillonella*	*Streptococcus*	*Veillonella*
3	***Haemophilus*** ^a^	***Haemophilus***	*Veillonella*	***Prevotella***
4	*Rothia*	*Fusobacterium*	***Moraxella***	***Haemophilus***
5	*Lactobacillus*	*Rothia*	*Rothia*	***Actinomyces***
6	***Prevotella***	***Prevotella***	***Prevotella***	*Rothia*
7	*Neisseria*	***Actinomyces***	***Actinomyces***	*Fusobacterium*
8	***Actinomyces***	*Neisseria*	*Megasphaera*	*Neisseria*
9	*Gemella*	*Lactobacillus*	*Lactobacillus*	*Gemella*
10	*Granulicatella*	*Porphyromonas*	*Neisseria*	*Leptotrichia*
11	*Fusobacterium*	*Gemella*	*Porphyromonas*	*Granulicatella*
12	*Leptotrichia*	*Granulicatella*	*Fusobacterium*	*Porphyromonas*
13	*Megasphaera*	*Leptotrichia*	*Alloprevotella*	*Megasphaera*
14	*Porphyromonas*	*Alloprevotella*	*Gemella*	*Alloprevotella*
15	*Alloprevotella*	*Megasphaera*	*Leptotrichia*	*Lactobacillus*
16	***Moraxella***	***Moraxella***		***Moraxella***

*Only genera that represent at least 1% of total sequences are shown

^*a*^Select clinically or statistically significant taxa are bolded for emphasis

Wilcoxon rank-sum permutation tests with Benjamini-Hochberg correction were used to test for significant differences in ASV counts between phenotypes. Among oral wash samples with an FDR < 0.10, none of the ASVs differed by exacerbation phenotype. Among sputum samples, we did not find statistically significant differences in *Haemophilus*, *Moraxella*, or *Streptococcus* relative abundance at the ASV or genus level, as only 3 of the 9 available FE sputum samples exhibited striking shifts in abundance of these taxa. We did find 12 less common sputum ASVs that differed by exacerbation phenotype (Table 5). *Actinomyces*, a gram-positive anaerobic organism capable of causing both oral and pulmonary infections had the most evidence for a difference. It was more common in the sputum of IE compared to FE. Since exacerbation phenotype and FEV1pp may be associated with each other in our dataset, we used FEV1pp as a co-variate in our model. *Actinomyces*, *Capnocytophaga*, *Gemella*, *Ruminococcaceae*, *Candidatus_Saccharimonas*, *Prevotella*, and *Bergeyella* ASVs all exhibited an FDR < 0.10 with respect to exacerbation phenotype, after correcting for FEV1pp. All ASVs were more abundant in IE sputum compared to FE sputum.

**Table 5:** Sputum Taxa Differential Abundance by Exacerbation Phenotype

Corresponding Genus for Identified ASV	*q*-value	*q*-value (corrected for FEV1pp^*a*^)	Difference in Group Means (all are more abundant in IE sputum than FE sputum)
***Actinomyces*** ^***b***^	**0.048**	**< 0.001** ^*b*^	169.6
***Capnocytophaga***	**0.087**	**< 0.001**	42.5
***Gemella***	**0.087**	**< 0.001**	2379.2
*Streptococcus*	0.087	0.382	500.8
*Campylobacter*	0.087	0.591	29.8
***Ruminococcaceae***	**0.087**	**0.057**	23.9
***Candidatus_Saccharimonas***	**0.087**	**0.019**	63.8
***Prevotella***	**0.087**	**0.066**	42.5
*Prevotella*	0.087	0.443	18.4
Lachnospiraceae	0.087	0.289	96.3
***Bergeyella***	**0.087**	**0.057**	36.6
*Treponema*	0.087	0.201	7.6

^*a*^FEV_1_ percent predicted

^*b*^ASVs with corrected q-values <0.10 are bolded for emphasis

## Discussion

Our study of frequent and infrequent COPD exacerbators is among the first comparisons of the oral and sputum microbiota based on exacerbation phenotype. COPD exacerbations are associated with bacterial infection as well as excess morbidity and mortality, and the exacerbation-associated lung microbiota is an area of active study. Few studies have compared the lung microbiota of FE and IE during periods of clinical stability. Here we have shown that the sputum of FE has lower alpha diversity than the sputum of IE even during periods of clinical stability and without recent administration of antibiotics and/or systemic corticosteroids. This finding is consistent with the general observation that lower alpha diversity is associated with worsening lung health. Although our data show significant within-subject similarity of the oral and sputum microbiota, this did not preclude the identification of shifts in sputum abundance of select taxa in association with exacerbation phenotype.

Differences in alpha diversity and taxa abundance between exacerbation phenotypes were primarily driven by the sputum samples. Oral wash samples alone did not appear to distinguish frequent from infrequent exacerbators. Although decreased alpha diversity and changes in taxa abundance correlated with exacerbation phenotype here, observational studies such as ours are unable to determine causation. Other clinical factors associated with exacerbation phenotype (medication use, obstruction severity, etc.) may be responsible for these associations between exacerbation phenotype and alpha diversity or taxa abundance. Although we excluded subjects who had received antibiotics in the last 1 month, it is likely that FEs received a greater number of antibiotic courses in the past year than were received by the IEs over the past year. Prior antibiotic exposure may have been responsible for some of the changes in alpha diversity between phenotypes noted here. Our facility favors the use of doxycycline to treat COPD exacerbations. Doxycycline is a tetracycline antibiotic, whose effects on the oral and gut microbiota were shown to be less persistent than the effects of other antibiotics like ciprofloxacin or clindamycin. [40] However, prior antibiotic exposure should have affected both the oral and sputum microbiota of our subjects. Our study identified greater phenotype-associated changes in the sputum compared to oral microbiota, suggesting that antibiotic-associated changes are unlikely to be responsible for all of the lung-specific changes noted here.

Although it may be hypothesized that FEs harbor a greater number of bacteria in their airways and sputum when compared to IEs, our data do not support this hypothesis. After normalizing 16S copy numbers to sample mass, sputum contained a larger number of 16S copies than did oral wash samples, regardless of phenotype. No differences in 16S copy numbers based on exacerbation phenotype were observed.

Our studies using alpha diversity demonstrate lower diversity in sputum samples compared to oral wash samples, with the lowest alpha diversity observed in the FE sputum samples. This is consistent with studies of other chronic lung diseases such as cystic fibrosis, in which lower alpha diversity is associated with more severe disease. Review of the taxa found in FE sputum samples demonstrates that three subjects (5, 19, and 22) exhibited a high relative abundance of Gammaproteobacteria (specifically *Haemophilus* and *Moraxella,* two organisms associated with COPD exacerbations), which corresponded to low alpha diversity scores. The pathogen dominance exhibited by these three FE sputum samples is in contrast to these subjects’ oral wash findings, which had higher diversity scores and did not exhibit pathogen dominance. Conversely, there were no IE sputum samples that demonstrated pathogen dominance and resulted in very low diversity scores. The three FEs with low sputum alpha diversity did not appear to differ from the FE group as a whole with respect to obstruction severity, number of exacerbations in the last year, or symptom severity. Our data are most consistent with the notion that even during exacerbation-free intervals, a minority of FEs have low alpha diversity due to colonization with potentially pathogenic organisms—and they are not readily distinguished from non-colonized FEs based on other clinical factors.

Although the FE sputum samples from subjects 5, 19, and 22 were different from these subjects’ oral wash samples, other FE and IE sputum-oral wash pairs exhibited a high degree of similarity in beta diversity analyses. Clustering analysis demonstrated that for 18 sputum samples, in nine instances the sputum sample’s nearest neighbor in the dendrogram was the oral wash sample from the same subject. Of these nine very similar sputum-oral wash sample pairs from the same subject, six sample pairs were from IE while three sample pairs were from FE. In contrast, sputum and oral washes from FE subjects 5, 19, and 22 did not cluster together in the dendrogram or the beta diversity plots. Despite the overall similarity in oral wash and sputum sample beta diversity within subjects, we did not detect a change in within-subject beta diversity related to exacerbation phenotype (as we may have expected based on the findings from FE subjects 5, 19, and 22). Additionally, oral wash alpha diversity appeared to be a predictor of sputum alpha diversity, further supporting the similarities we saw between subjects’ oral and sputum microbiota. While there is likely a correlation between the sputum and oral wash microbiota from the same subject, additional exploration of the potential role of exacerbation phenotype in this relationship is warranted.

PERMANOVA analyses identified significant clustering based on anatomic site sampled, exacerbation phenotype, COPD severity, time since last professional dental cleaning, current tobacco use, and inhaled corticosteroid use. Due to our sample size of 42, these analyses were performed for all samples simultaneously, rather than separate analyses of oral wash and sputum samples. Although the literature suggests that COPD severity clustering is more likely due to sputum microbiota changes and clustering based on dental habits or tobacco use is more likely driven by the oral microbiota changes, due to our small sample size we did not evaluate which sample site or taxa were the primary drivers of clustering. Time since last professional dental cleaning, COPD severity, and anatomic site appeared to have a greater influence on beta diversity than tobacco use, phenotype, or ICS use.

Our analysis of taxa distribution across anatomic sites and phenotypes identified shifts in the most abundant taxa in FE sputum samples compared to IE sputum samples, although the increases in *Haemophilus* and *Moraxella* abundance in FE sputum did not reach statistical significance. The most likely explanation for this lack of statistical significance is that increases in these two taxa were only noted in three of nine FE sputum samples. *Actinomyces* was significantly more abundant in IE sputum compared to FE sputum, and this result persisted after controlling for obstruction severity (FEV1pp). Increased relative abundance of *Actinomyces* in the sputum microbiota was also recently associated with survival following hospitalization for COPD exacerbation. [41] While *Actinomyces* is a common and clinically-relevant oral and lung pathogen, we currently lack robust evidence to assert a protective effect on the microbiome in COPD. It is possible that *Actinomyces* in the COPD lung provides protective benefits unrelated to other organisms, or that *Actinomyces* helps eliminate other more pathogenic members of the COPD microbiota, or simply that its relative abundance in the lung appears to increase when harmful and abundant COPD-associated taxa (such as *Moraxella* or *Haemophilus*) are not present. Additional studies will be needed to differentiate among these possibilities. Of note, oral wash sample taxa abundance did not appear to be correlated with exacerbation phenotype. This is consistent with exacerbation phenotype primarily influencing the lung (as opposed to oral) microbiota and suggests that phenotype-associated effects are not solely related to differences in antibiotic exposure between phenotypes.

We examined and compared oral wash samples to sputum samples in this study for several reasons. Firstly, we wished to compare the two sites to determine if sputum was or was not too heavily contaminated by oral bacteria during expectoration to identify phenotype-associated factors. Our data show that oral contamination of sputum samples has not prevented us from identifying phenotype-associated sputum microbiota changes. Secondly, we wished to address the possibility that differences in antibiotic exposure between phenotypes are responsible for the associations we identified. While antibiotic use should affect both the oral and sputum microbiota, the changes we observed in the sputum (but not oral) microbiota support the hypothesis that the lung microbiota changes we observed were due to phenotype-associated factors in the lung rather than systemic factors such as antibiotic use. In addition, the oral microbiota is the source of the lung microbiota through microaspiration or direct mucosal dispersion. The two sites are closely related and therefore the inclusion of oral samples in this study enhances our understanding of the COPD lung microbiota.

Our study had several strengths. We were able to study subjects during periods of clinical stability (at least 1 month after exacerbation treatment had ended), when their oral and sputum microbiotas had reached a period of relative clinical stability and were less likely to be significantly influenced by recent antibiotic or systemic corticosteroid exposure. In spite of the similarities between subjects’ oral and sputum microbiotas, we were able to observe differences in sputum taxa abundance and overall beta diversity based on exacerbation phenotype. We suggest that exacerbation phenotype, rather than increased antibiotic exposure among FEs, is responsible for the observed sputum microbiota changes. If increased antibiotic use among FEs had been entirely responsible for the exacerbation phenotype-related changes we observed in the sputum microbiota, we should also have observed similar phenotype-related changes in the oral microbiota. Both sites should be affected by antibiotic exposure.

Although our study successfully achieved our goals, it also had several weaknesses. Most importantly, this study is unable to determine if sputum microbiota changes caused the development of the FE phenotype or if the FE phenotype caused the sputum microbiota changes. Separating cause from effect in this instance would require identifying and sampling subjects prior to development of the FE or IE phenotype or significant antibiotic exposure. As in any non-randomized study, it is possible that factors other than exacerbation phenotype are responsible for the changes observed here. In addition, the degree of similarity between the oral and the sputum microbiota may suggest that sputum is too contaminated by oral taxa to be a reliable indicator of the lung microbiota. This concern is unlikely to have significantly affected our results as we were able to observe several alterations in the sputum microbiota correlated with exacerbation phenotype, and the effect of oral contamination would have been to mask sputum differences between phenotypes. There was a non-significant trend towards more significant lung obstruction in the FE group compared to the IE group. Therefore, some of the observed differences between phenotypes may be related to increased obstruction. However, many of our taxa abundance results remained significant following statistical correction for obstruction severity. Due to our sample size, we were also unable to determine if clustering on beta diversity analyses was primarily the result of differences between oral or sputum samples and we may have been underpowered to detect some differences, particularly between exacerbation phenotypes.

## Conclusions

We showed that prospectively-identified, clinically-stable frequent exacerbators have lower upper airway alpha diversity than infrequent exacerbators, and this finding may be driven by a minority of frequent exacerbators whose sputum is dominated by Gammaproteobacteria (*Moraxella, Haemophilus*). We identified differences in taxa relative abundance between phenotypes in sputum but not oral wash samples, and many of these differences persisted after correction for severity of obstruction. The upper airway microbiota of COPD subjects demonstrated clustering based on COPD exacerbation phenotype, inhaled corticosteroid use, tobacco use, severity of obstruction, and time since last professional dental cleaning. Dental care habits, tobacco cessation, and inhaled corticosteroid use are all modifiable factors and potential future targets for improving lung health via alterations in the upper airway microbiota.

## Additional file

Additional file 1: Supplementary information. (DOCX 2881 kb)

## Data Availability

The datasets generated during the current study are publicly available at NCBI Sequence Read Archive (SRA) under BioProject #543785: http://www.ncbi.nlm.nih.gov/bioproject/543785.

## Abbreviations

ANOVA: analysis of variance
ASV: amplicon sequencing variant
BMI: body mass index
COPD: Chronic obstructive pulmonary disease
FDR: false discovery rate
FE: frequent exacerbator
FEV1pp: forced expiratory volume in 1 s percent predicted
ICS: inhaled corticosteroid use
IE: infrequent exacerbator
PERMANOVA: permutational multivariate analysis of variance
SGRQ: St. George’s Respiratory Questionnaire
SILVA: (rRNA gene database named after the Latin word for forest)
