# Supplementary material for: Chronic obstructive pulmonary disease upper airway microbiota alpha diversity is associated with exacerbation phenotype: a case-control observational study
Source: Respir Res. 2019 Jun 7;20:114. doi: 10.1186/s12931-019-1080-4 (PMC6555967; doi:10.1186/s12931-019-1080-4)
Supplement: Supplementary file 1 — Supplementary information. (DOCX 2881 kb) [file 12931_2019_1080_MOESM1_ESM.docx]

Supplemental Information

**Methods**

**Subjects.**

**Table 1S. Study Inclusion and Exclusion Criteria.**

| Frequent Exacerbators | Inclusion Criteria | Exclusion Criteria |
| --- | --- | --- |
|  | 1. Age ≥ 40 years | 1. Use of antibiotics or oral corticosteroids in the last 1 month |
|  | 2. FEV_1_/FVC ≤0.7 | 2. **COPD exacerbation in the last 1 month** |
|  | 3. FEV_1_ ≤ 70% predicted | 3. Asthma diagnosis |
|  | 4. Current or former smoker with a ≥10 pack-year history of tobacco use | 4. Malignancy |
|  | 5. **≥ 1 exacerbation in the last 12 months** | 5. History of lung lobectomy |
|  |  | 6. Use of supplemental oxygen |
| Infrequent Exacerbators | Inclusion Criteria | Exclusion Criteria |
|  | 1. Age ≥ 40 years | 1. Use of antibiotics or oral corticosteroids in the last 1 month |
|  | 2. FEV_1_/FVC ≤ 0.7 | 2. **COPD exacerbation in the last 24 months** |
|  | 3. FEV_1_ ≤ 70% predicted | 3. Asthma diagnosis |
|  | 4. Current or former smoker with a ≥10 pack-year history of tobacco use | 4. Malignancy |
|  |  | 5. History of lung lobectomy |
|  |  | 6. Use of supplemental oxygen |

**Sample acquisition.** Oral samples were obtained by having subjects swish sterile water in their mouths for 30 seconds and then expectorating into a DNA-free tube. Sputum was induced using 3% saline (0.9% saline if FEV_1_ <40% predicted) and collected in a DNA-free container. Three subjects (2 IE and 1 FE) were unable to provide a sputum sample. All samples were weighed and frozen at -80°C until sample processing. DNA contamination of reagents and equipment was evaluated using extraction controls, which were processed and analyzed alongside the experimental samples. Negative control samples consisted of unused sterile water placed in oral wash tubes and processed alongside samples.

**DNA extraction and 16S rRNA gene sequencing.** After thawing, sputum was treated with sputolysin and incubated at 37°C for 15 minutes prior to pelleting and resuspension in MO BIO PowerSoil DNA Isolation Kit buffer (QIAGEN, Germantown, MD). Oral wash and negative control samples were thawed, pelleted, and resuspended in power bead tube buffer solution (QIAGEN). Once resuspended, both sample types underwent DNA isolation according to the MO BIO PowerSoil DNA Isolation Kit protocol. 16S rRNA V4 amplicons were generated via 20 PCR amplification cycles using primers 515F and 806R. Amplicons were sequenced with an Illumina MiSeq 600 cycle v3 kit using paired-end reads at the University of Minnesota Genomics Center.

Negative control analysis. Negative controls consisting of unused sterile water were placed in sterile oral wash tubes and analyzed as a negative control for both oral wash and sputum samples. Sputum samples were expectorated into the same sterile tubes as the oral washes. Due to the unavoidable presence of bacterial DNA even in sterile materials, negative control samples carried a very small amount of background contamination. By examining our 16S rRNA quantification results (Figure 2), we can see that our oral wash samples contained ~10,000 times more bacterial DNA than our negative control samples. Our sputum samples contained ~100,000-1,000,000 times more bacterial DNA than our negative control samples. Our sequencing results yielded a significantly greater number of sequences from our samples than our controls, as described in the Methods section of the main manuscript. To control for different sampling depth across samples and eliminate samples that did not sequence well, we subsampled all samples to 25,955 sequences. This eliminated all negative control samples (all of which contained 732 or fewer sequences) and one frequent exacerbator sputum sample that produced 17,567 sequences. Please see supplementary figure 1 for a beta-diversity plot containing both negative controls and samples. Using the conservative estimate provided above by our quantitative PCR studies, for every 1 contaminating bacterial sequence in our starting materials, we should expect 10,000 true bacterial sequences in the oral wash samples and 100,000-1,000,000 true bacterial sequences in the sputum samples.

**Quantitative PCR (qPCR).** To determine 16S rRNA gene copy numbers, qPCR was performed in triplicate for all samples and controls. Twenty μl reactions using 16S rRNA qPCR primers 338-F (5’-ACTCCTACGGGAGGCAGCAG-3’) and 518-R (5’-ATTACCGCGGCTGCTGG-3’) at a final concentration of 0.67 μM for each primer. The LightCycler 480 SYBR Green I Master Kit (Roche) was utilized for qPCR on the Roche LightCycler 96. Cycling conditions were 50**°**C for 2 min, 95**°**C for 2 min, then 40 cycles of 95**°**C for 15 sec, 58**°**C for 15 sec, and 72**°**C for 30 sec, followed by a melting curve. The standard curves for absolute quantification of 16S rRNA gene copy numbers were constructed using the DH5α *E. coli* strain by initially creating an end-point PCR product of the DH5α strain with universal 16S rRNA gene primers Bact-27F and Bact-1492R [1]. The standard curve was created using ten-fold serial dilutions of the *E. coli* PCR product. Copy numbers were normalized to sample mass.

**Data processing.** Sputum samples may be contaminated by oral taxa during expectoration. Unfortunately, there is no consensus on how to identify and remove oral contaminant taxa from true sputum taxa; any potential method has the potential to arbitrarily and significantly skew the microbiota samples based on the parameters chosen when “subtracting” contaminant taxa. Many taxa are true members of both microbiota, complicating efforts to remove these taxa or a portion of these taxa. In the absence of a consensus among investigators, attempts to subtract oral contamination from the sputum microbiota may lead to the unintentional introduction of a systematic bias in our dataset. While such a procedure is logical to consider, we have not performed this analysis here as we feel the chance of introducing bias into our dataset is too high. We do not feel that oral contamination of sputum samples during expectoration has prevented us from obtaining valuable information from our dataset. The effect of commingled oral taxa in sputum samples would have been to dilute differences in the sputum observed between phenotypes. This was not a significant concern in our dataset. We identified significant changes in sputum associated with exacerbation phenotype (see Figure 3 and Tables 4 and 5).

**Statistical Analysis**. Subject characteristics were analyzed with a Fisher-Pitman permutation test for continuous variables and Fisher’s exact test for categorical variables. ANOVA analyses were performed to compare 16S copy numbers across sites and post-hoc pairwise permutation testing was performed with Bonferroni-Holm correction (0.05 level). A linear mixed model (with subject included as a random effect) with Bonferroni-Holm correction was used to evaluate for associations between 16S copy numbers, phenotype, and sampling site. *P*-values were obtained using a 2-sided permutation test modeling site, phenotype, and patient as a random effect with 1,000 permutations. To determine clinical factors associated with α-diversity, linear mixed models with diversity score as a response, patient as a random effect, and site, phenotype and their interaction as fixed effects were employed. Confidence intervals are reported for linear model estimates and results from a mixed effects linear model. *P*-values were obtained using a 2-sided permutation test with 1,000 permutations modeling alpha diversity using phenotype, site, phenotype:site interactions and patient-level random effects. To determine clinical factors associated with β-diversity, PERMANOVA testing with default parameters using anatomic site, phenotype, inhaled corticosteroid (ICS) use, current smoking, COPD severity, time since last professional dental cleaning, and alcohol use was performed on the Bray-Curtis dissimilarity matrix. Within-subject dissimilarity was calculated by taking the mean of the observed dissimilarity between each subject’s oral and sputum samples. A permutation test was performed by randomly permuting subject labels (100,000 times) and recalculating the mean dissimilarity across all sample pairs. The mean dissimilarity from these permutations provides an estimate of the between-subject dissimilarity. A *p*-value for this permutation test was estimated with the proportion of times that the observed within-subject dissimilarity is more extreme than the dissimilarity between oral and sputum samples from different subjects (a 1-sided test). This analysis was performed for all subjects, and then separately for FEs and IEs. Wilcoxon rank-sum permutation tests were used to determine differences in ASV counts between phenotypes. Tests were performed separately for each ASV and the process was performed separately for each anatomic site. Results were also assessed after controlling for FEV_1_ percent predicted (FEV1pp), a potential co-linear clinical variable with phenotype. In ASV analyses, the Benjamini-Hochberg procedure was used to control the false discovery rate at 0.10. ASV count data was used to cluster samples with complete linkage hierarchical clustering using hclust function in base R. All analyses were performed in R version 3.4.2.

**Supplementary Table S2. Subject Characteristics for Subjects with Evaluable Sputum**

|  | **Infrequent Exacerbator** | **Frequent Exacerbator** | **p value*** |
| --- | --- | --- | --- |
| N | 9 | 9 |  |
| Sex, Male (%) | 9 (100) | 9 (100) |  |
| Age, mean (sd) | 67.44 (4.61) | 69.56 (7.11) | 0.45 |
| Race, white (%) | 9 (100) | 8 (88.9) | 1.00 |
| BMI, mean (sd) | 29.54 (6.17) | 26.39 (7.56) | 0.33 |
| Hypertension, Yes (%) | 5 (55.6) | 5 (55.6) | 1.00 |
| Diabetes, Yes (%) | 2 (22.2) | 3 (33.3) | 1.00 |
| COPD Severity (%) |  |  |  |
| Moderate | 3 (33.3) | 3 (33.3) |  |
| Severe | 4 (44.4) | 1 (11.1) |  |
| Very Severe | 2 (22.2) | 5 (55.6) |  |
| FEV_1_ % predicted | 44.78 (14.65) | 34.56 (16.76) | 0.18 |
| COPD exacerbations in the last 12 months, mean (sd) | 0 (0) | 3.00 (1.12) | <0.001 |
| COPD hospitalizations in the last 12 months, mean (sd) | 0 (0) | 0.56 (0.73) | 0.04 |
| Inhaled corticosteroid use, Yes (%) | 6 (66.7) | 5 (55.6) | 1.00 |
| Pack-years of smoking, mean (sd) | 57.22 (31.11) | 59.22 (33.21) | 0.88 |
| Current tobacco use, Yes (%) | 4 (44.4) | 2 (22.2) | 0.62 |
| Current alcohol use, Yes (%) | 6 (66.7) | 7 (77.8) | 1.00 |
| Brush teeth ≥ once daily (%) | 7 (77.8) | 8 (88.9) | 1.00 |
| History of periodontal disease, Yes (%) | 2 (22.2) | 2 (22.2) | 1.00 |
| SGRQ Score, mean (sd) | 49.20 (8.53) | 49.08 (14.68) | 0.98 |

BMI, body mass index; COPD, chronic obstructive pulmonary disease; FEV1 % predicted, forced expiratory volume in 1 second, percent of predicted value; sd, standard deviation; SGRQ, St. George’s Respiratory Questionnaire.

*A Fisher-Pitman permutation test was conducted for all continuous variables and a Fishers exact test for all categorical variables.

**Supplemental Figures**

**Supplemental Figure 1.**

**
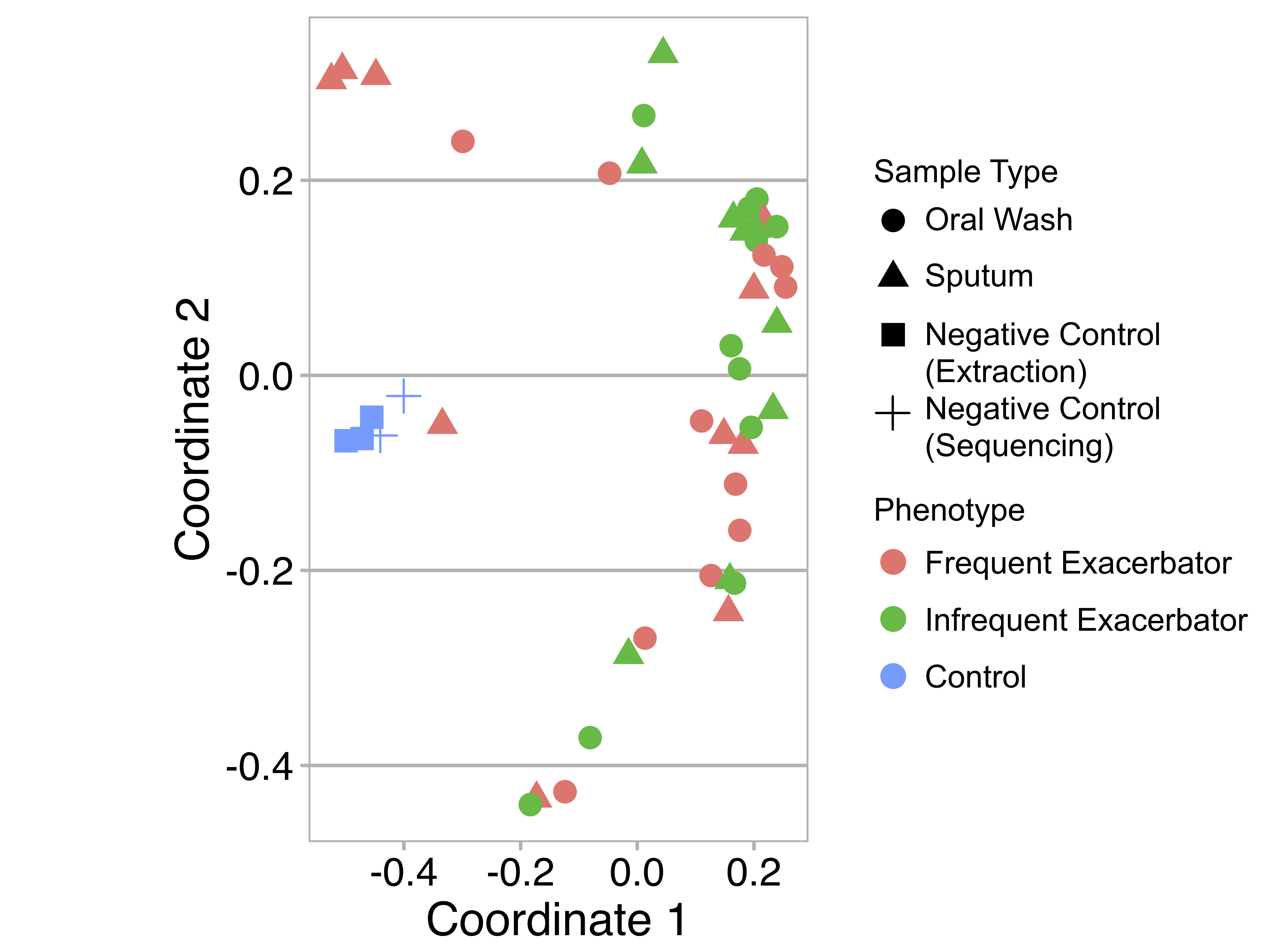
**

**Subject Samples Are Distinct From Negative Controls**. Principal coordinate analysis using Bray-Curtis dissimilarity was plotted with color used to define subject phenotype or control status and shape used to define sample type. Negative control samples (in blue) consisted of both extraction controls (squares) and sequencing center controls (+). The control samples clustered closely and were distinct from almost all subject samples.

**Supplemental Figure 2.**


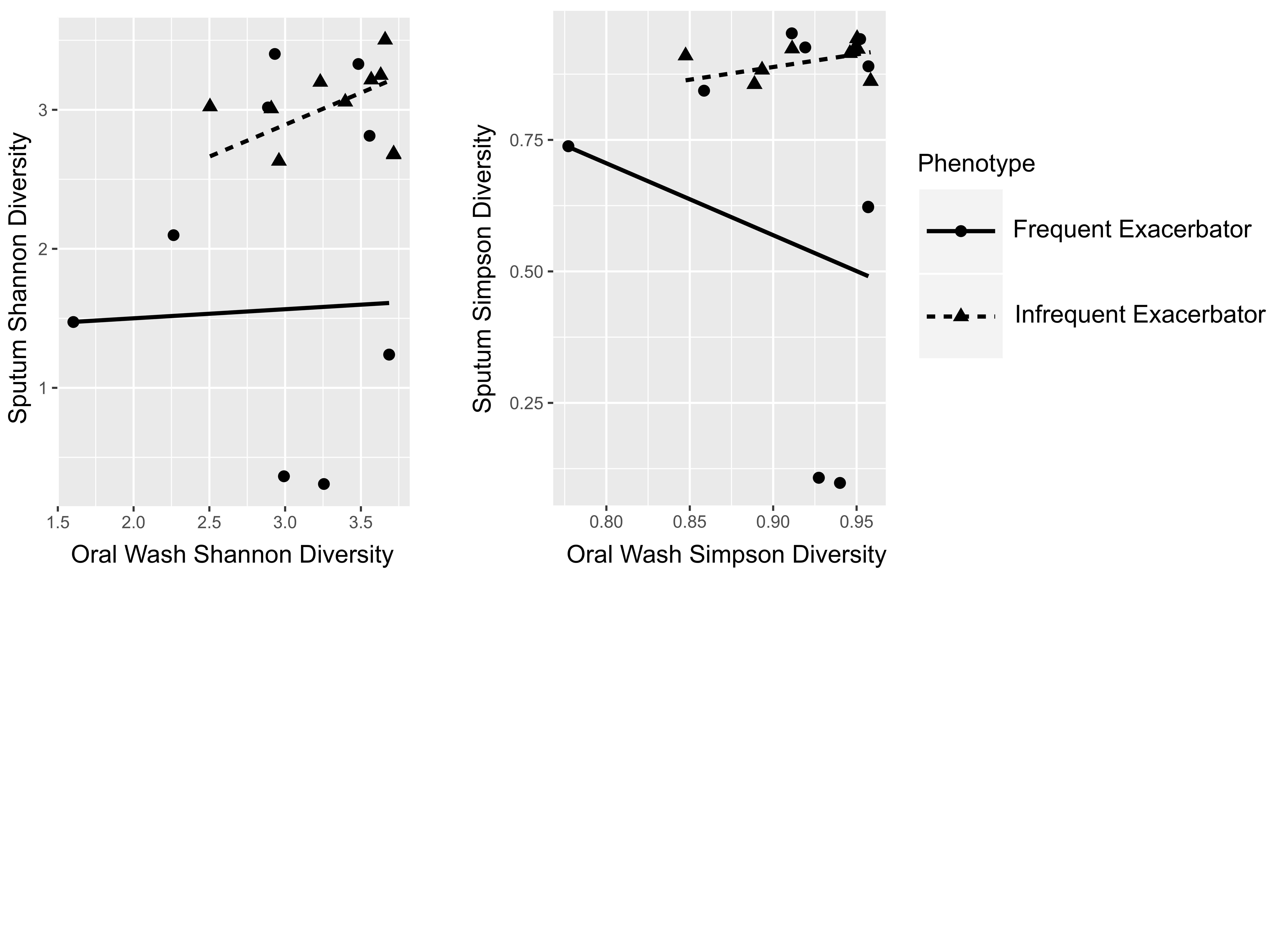


**Sputum Alpha Diversity And Oral Wash Alpha Diversity Models Are Not Robust To Removal Of Low Diversity X-Outlier.** After removal of a low-diversity x-outlier from the dataset, the linear models were re-run. Circles represent FE samples while triangles represent IE samples. Linear models were used to predict sputum alpha diversity based on oral wash alpha diversity. Oral wash Shannon diversity was a significant predictor of sputum Shannon diversity (*p* = 0.039), but after incorporating phenotype and the interaction of oral wash Shannon diversity and phenotype all factors were non-significant. Oral wash Simpson diversity (alone or in combination with phenotype and its interaction) was not a significant predictor of sputum Simpson diversity. The dotted lines represent a linear model of IE samples alone, while the solid lines represent a linear model of FE samples alone. While phenotype was not a significant predictor in either model, IE appeared to have a more positive linear relationship between sputum and oral wash samples than did FE.

**References**

1. Huang YJ, Nelson CE, Brodie EL, Desantis TZ, Baek MS, Liu J, et al. Airway microbiota and bronchial hyperresponsiveness in patients with suboptimally controlled asthma. J Allergy Clin Immunol*.* 2011;127:372-381.e1.
